# Associations with oral health indices for obesity risk among Japanese men and women: results from the baseline data of a cohort study

**DOI:** 10.1186/s12889-022-13998-w

**Published:** 2022-08-22

**Authors:** Hiroko Tanaka, Mirei Nakano, Kiyonori Kuriki, Shizuoka-Sakuragaoka J-MICC Study Group

**Affiliations:** Laboratory of Public Health, Graduate School of Integrated Pharmaceutical and Nutritional Sciences, University of Shizuoka, 52-1 Yada, Suruga-ku, Shizuoka, 422-8526 Japan

**Keywords:** Oral health index, Oral self-care habits, Oral hygiene, Oral function, Mastication ability, Body mass index, Obesity risk, Adults

## Abstract

**Background:**

Oral health is composed of various oral health indices (OHIs), such as oral self-care habits, oral hygiene, oral function, and mastication ability. Oral self-care habits have frequently been examined for obesity risk. This study aimed to comprehensively clarify the association between OHIs and obesity risk.

**Methods:**

We collected data for 15 questions on the four OHIs and measured the body mass index of 3494 men and 2552 women aged 35–79 years. Obesity was defined as a body mass index ≥25 kg/m^2^. The four OHIs were scored by the corresponding questions (good as “reference”), and the summed score was defined as “comprehensive OHI”, that is, the fifth OHI. Each lowest tertile score was used as “reference”. Using multiple logistic regression analysis, odds ratios (ORs), 95% confidence intervals (CIs), and *p*-values for trends were estimated.

**Results:**

In the men and women, the ORs were 1.37 (1.11–1.67, < 0.01) and 2.48 (1.80–3.42, < 0.01) for oral self-care habits, and 1.78 (1.42–2.24, < 0.01) and 3.06 (2.12–4.43, < 0.01) for tooth brushing frequency, respectively. Moreover, in men, a significant trend was found for “harder rinsing out your mouth”, related to “oral function”. In women, the ORs were 1.74 (1.28–2.36, < 0.01) and 1.43 (1.00–2.06, < 0.01) for “comprehensive OHI” and “longer meal time” related to “mastication ability”, respectively.

**Conclusions:**

Our findings showed that obesity risk was associated with poor of oral health, which were comprehensively composed of various OHIs, among middle-aged and older Japanese men and women.

## Background

According to the World Health Organization (WHO) and FDI World Dental Federation, oral health is multifaceted and consists of a variety of oral health indices (OHIs) such as oral self-care habits, oral hygiene, oral function, and mastication ability [1, 2]. Oral health does not only good teeth, but it also is integral to general health and essential for well-being; therefore, it is thought to be a risk factor for non-communicable diseases (NCDs), such as diabetes and cardiovascular disease, which are induced by obesity [3]. In previous studies, however, oral health for the risks of NCDs and obesity has not been comprehensively examined by OHIs among middle-aged and older people.

Among the target population for preventing NCDs, OHIs such as periodontal pockets and periodontitis are used in dental practice, and the community periodontal index is applied to dental health in individual communities. Although few studies have evaluated periodontal pockets and the community periodontal index in community population because of the complicated clinical examination required, such studies have reported that those with good oral health have a lower risk of NCDs [4]. However, the remaining teeth are important for preventing malnutrition and frailty among older people [5], and the association with the risk of NCDs has been investigated in many epidemiological questionnaire surveys. Surprisingly, most have reported a higher risk of obesity in older people with fewer remaining teeth because they often swallow without chewing well [6–8]. To prevent gum disease, tooth decay, and tooth loss, dental guidance and oral health care are encouraged; residents are directed to perform tooth brushing and increase the frequency of tooth brushing. A specific OHI related to oral self-care habits has been used to evaluate the risk of NCDs among middle-aged and older adults [9–11]. In Western countries, negative associations with tooth brushing frequency have been found for risks of diabetes, cardiovascular diseases, and hypertension [12, 13]. In Japan, such an association has been shown for obesity risk [14].

In Japan, a variety of OHIs have been reported by annual governmental surveys on dental diseases [15]. The proportion of people who brush their teeth more than twice daily has been increasing over the past 40 years, with more in women than in men and declining with age. Age-specific differences were observed for higher proportions of the following OHIs: “toothache/sensitive tooth” and “gingival pain/swelling/bleeding” as oral hygiene among middle-aged people, and “difficulty chewing some foods” as oral function among older people. The National Health and Nutrition Survey has shown that 90% of those aged 50–59 years, 70% of those aged 60–69 years, and 45% of those aged 70–79 years have more than 20 teeth [16]. The survey also reported that those with a body mass index (BMI) ≥25 kg/m^2^ increased in men aged 30–39 years and was the highest in men aged 50–59 years, while it increased in women aged 40–49 years and was highest in women aged 60 years and older.

Many Japanese studies have reported a positive relationship between eating rate and obesity risk as eating behavior [17–19], but unfortunately not between oral functions (i.e., occlusal forces) and mastication ability. To date, therefore, few studies have reported any associations between various OHIs and obesity risk. Based on the results of previous studies examining the associations with some specific limited OHIs, our hypothesis of this study is that poor oral health is associated with obesity risk with reference to the WHO’s oral health, which comprehensively assesses various OHIs. Among middle-aged and older people, the present study examined obesity risk according to multiple OHIs, including oral self-care habits, oral hygiene, oral function, and mastication ability.

## Methods

### Participants of the study

The purpose and outline of the Japan Multi-Institutional Collaborative Cohort (J-MICC) Study was to prospectively investigate gene-environment interactions for the risk of NCDs, as described elsewhere [20]. In brief, approximately 100,000 study subjects aged 35–69 years participated in the baseline survey, and the J-MICC Study was executed using a systematic research protocol for all 13 research sites. Each collaborative research site was designed using a common protocol reflective of the J-MICC Study and additional original protocols were used. The study was performed over 20 years of follow-up to examine health outcomes. As part of the Shizuoka Sakuragaoka J-MICC Study [17], which was added to our regional research protocol at this site, the current cross-sectional study was conducted among 6395 men and women aged 35–79 years. Written informed consent was obtained from each participant after explaining the outline and purpose of the two study designs. All the research procedures were conducted in accordance with the Declaration of Helsinki and were approved by the Ethics Committee of Nagoya University Graduate School of Medicine (No.2010–0939-8), Aichi Cancer Center Research Institute (No.H2210001A), and University of Shizuoka (No.22–39). The participation rate was 27.6% for all participants. In the current study, 3494 men and 2552 women were finally selected as eligible participants after excluding the following criteria: 1) missing data on OHIs and measured body weight and height (*n* = 52), and 2) missing data on lifestyle information used as potential confounding factors (*n* = 231).

### Definitions of obesity

During the health checkups, body weight and height were measured by trained nurses at five health checkup centers. The individual body mass index (BMI) was calculated by dividing the participants’ weight (kg) by the square of their height (m). Obesity is defined as a BMI ≥25 kg/m^2^ according to the Japan Society for the Study of Obesity (JASSO) [21], in consideration of WHO expert comments [22]. In this study, subjects with a BMI ≥25 and < 25 kg/m^2^ were defined as obese and non-obese group, respectively. The rationale for this study is that, compared to European populations, Asian populations have been shown to be at a higher risk for NCDs, even when BMI is lower than the existing WHO cutoff points for overweight and obesity. According to the guidelines for the management of obesity disease 2016 by JASSO [23], however, obesity should be diagnosed only after a physician examines the patient and comprehensively determines whether the patient has any health conditions or excessive accumulation of visceral fat.

### Data collection of lifestyle information

The self-administered questionnaire consisted of both common (e.g., conventional lifestyle, medical history, and general tooth/gum conditions including tooth number) and original items (e.g., OHIs and dietary behaviors such as snacking) from all 13 research sites. Briefly, we collected the following data from each participant: physical activity, smoking status, habitual drinking, and snaking as a conventional lifestyle; medications for diabetes, dyslipidemia, hypertension, angina myocardial infarction, and cerebral stroke as medical history; tooth pain, gum bleeding, and gum swelling as general tooth/gum conditions; and age, education level, and tooth number as others. A scientifically validated short food frequency questionnaire (FFQ) was used to assess habitual diet, including total energy intake, and we have also reported success with the use of FFQ at our site using data from our site [24–27]. The validity and reproducibility tests for the selected foods have already been reported as follows: almost 0.50, as the median de-attenuated Spearman’s rank correlation coefficient between dietary records (for a total of 12 days over four seasons) and the first FFQ, and > 0.60 as the median energy-adjusted Spearman’s rank correlation coefficients between the first FFQ and the second FFQ. Physical activity and exercise were calculated as “Mets‧h/day” [28]. Trained scientific nurses reviewed the questionnaires to reduce missing data whenever possible. Two or three additional checks were performed by other trained nurses, each using a blue and red ink pen.

### Definition of oral health indices

We used an oral health questionnaire with 15 questions administered to several municipalities [29]. In user’s guideline of the questionnaire, four OHIs of oral self-care habits, oral hygiene, oral function, and mastication ability were defined as follows: “tooth brushing frequency”, and “use of inter-dental care device” as oral self-care habits; “worried about dry mouth”, “sometimes food may be left in your mouth”, “a white moss-like substance is coating on your tongue”, and “worried about your own bad breath” as oral hygiene; “it is getting harder to rinse out your mouth”, “your voice has become hoarse”, “you can talk well”, “sometimes choking on tea or soup”, “you may choke during meals”, and “you may find it difficult to swallow food” as oral function; and “compared to six months ago, it is difficult for you to eat tough food”, “mealtimes are now longer than before”, and “sometimes you cannot taste it” as mastication ability, respectively. Fifteen items were answered on three levels (good/normal/poor), with the exception of two levels (good/poor) on items second, third, tenth, and thirteenth items. The four OHIs were scored on each composed question (good as “reference”), and also defined as “comprehensive OHI”, that is, the fifth OHI, according to their total score.

### Statistical analyses

Data were analysed using R version 3.6.1. All reported probability values were two-tailed and the level of significance was set at *p* < 0.05. All analyses were performed separately for men and women. As appropriate, t-tests and chi-squared tests were used for continuous and categorical nominal variables, respectively. The three and two levels of the 15 questions according to the oral health questionnaire were scored with ordinal numbers (i.e., 1, 2, and 3 points) representing “≥3 times/day, twice/day, and ≤once/day”, and “good, normal, and poor”, and those (i.e., 1 and 3 points) for yes and no. Poor oral health was defined as higher scores on each of the five OHIs. Each poor level of OHIs and their composed questions is postulated to be related to higher risks of NCDs, acceleration of aging and deduction of social participation, and ultimately to inhibition of prolonging healthy life expectancy [30].

Multivariable adjusted logistic regression analysis was used to estimate the odds ratios (ORs) and 95% confidence intervals (CIs) for obesity, compared with each 15 question on oral health (good as “reference”) and each five OHI (the lowest tertile as “reference”). With reference to previous studies [6, 14, 31], the potential confounding factors were applied as follows: age (years), physical activity (METs‧h/day), total energy intake (kcal/day), and tooth number (n) as continuous variables, and current smoker (no/yes = 0 / 1), current habitual drinker (no/yes = 0 / 1), snacking (none/sometimes/everyday = 1 / 2 / 3), education level (< 12 / 12 / > 12 years = 1 / 2 / 3), medications [no/yes = 0 / 1 for diabetes, dyslipidemia, hypertension, angina myocardial infarction, and cerebral stroke, respectively] and tooth/gum conditions [no/sometimes/yes = 1 / 2 / 3 for tooth pain, gum bleeding, and gum swelling, respectively] as categorical variables.

Using the scores described above as Model 1, trend associations were assessed with the median of each tertile score for the five OHIs. In Model 2, the OHI scores were recalculated by applying “1, 3, 5” and “ 1 and 5” points for the three and two levels of the questions comprised the OHI. Trends were calculated in the same way and compared with the results of Model 1 to check 1) whether the positive or negative β values were the same in Model 2, and 2) whether their statistical significances were also the same.

## Results

In men, Table 1 shows that the proportion of obese participants was 24.3%, and BMI of obese group was 27.3 ± 2.3 kg/m^2^ (*p* < 0.001). The obese group were slightly younger (*p* < 0.001). No difference was found in the number of remaining teeth and proportions of tooth pain, gum bleeding and gum swelling between the obese and non-obese group. The scores on the two OHIs, “oral self-care habits” and “comprehensive OHI” were higher in obese group rather than in non-obese group, and proportions of the two question “tooth brushing frequency” and “it is getting harder to rinse out your mouth” on “oral self-care habits” and “oral function”, respectively, were different between obese and non-obese group (at least *p* < 0.05 for all).

**Table 1:** Characteristics of male study participants (*n* = 3494)

Variables^a^	Obese group	Non-obese group	*p* value^b^
	(*n* = 850, 24.3%)	(*n* = 2644, 75.7%)	
Age (years)	56.2 ± 10.6	58.9 ± 10.8	< 0.001
Body mass index, BMI (kg/m^2^)	27.3 ± 2.3	21.9 ± 1.9	< 0.001
Lifestyle information			
Physical activity (METs‧h/day)	21.6 ± 15.4	24.0 ± 15.3	< 0.001
Current smoker (%)	25.4	24.4	0.58
Current habitual drinker (%)	73.1	76.7	< 0.05
Snacking, None / Sometimes / Everyday (%)	35.5 / 48.5 / 16.0	40.5 /45.4 / 14.1	< 0.05
Dietary information			
Total energy intake (kcal/day)	1964 ± 377	1996 ± 402	< 0.05
Education level, > 12 / 12 / < 12 years (%)	48.2/ 43.9 / 7.9	43.9 / 45.3 / 10.7	< 0.05
Receiving medications (%)			
Diabetes	15.2	8.9	< 0.001
Dyslipidemia	24.8	14.6	< 0.001
Hypertension	38.4	26.0	< 0.001
Angina myocardial infarction	5.4	3.6	< 0.05
Cerebral stroke	3.3	1.9	< 0.05
Information on tooth and gum			
Tooth number (n)	24.6 ± 6.0	24.3 ± 6.3	0.34
Tooth/gum condition, No / Sometimes / Yes (%)			
Tooth pain	58.9 / 21.3 / 19.8	60.2 / 21.6 / 18.2	0.59
Gum bleeding	63.9 / 23.1 / 13.1	65.6 / 23.6 / 10.8	0.20
Gum swelling	68.6 / 21.2 / 10.2	72.2 / 19.0 / 8.8	0.12
**Oral health indices (OHIs) and their composed questions**^**c**^			
**Oral self-care habits (score range: 2 to 6 points)**	4.54 ± 1.26	4.34 ± 1.31	< 0.001
Tooth brushing frequency, ≥3 times/day / Twice/day / ≤once/day (%)	18.6 / 42.9 / 38.5	24.9 / 44.1 / 31.1	< 0.001
Use of inter-dental care device	32.7 / 67.3	35.9 / 64.1	0.10
**Oral hygiene (score range: 4 to 12 points)**	5.51 ± 1.56	5.42 ± 1.55	0.14
Worried about dry mouth.	82.0 / 18.0	84.9 / 15.1	0.05
Sometimes food may be left in your mouth.	88.2 / 8.9 / 2.8	86.0 / 10.7 / 3.2	0.26
A white moss-like substance is coating on your tongue.	80.6 / 14.0 / 5.4	81.0 / 14.1 / 4.9	0.85
Worried about your own bad breath.	42.7 / 39.1 / 18.2	47.0 / 35.5 / 17.5	0.08
**Oral function (score range: 6 to 18 points)**	7.62 ± 1.95	7.57 ± 1.91	0.47
It is getting harder to rinse out your mouth.	91.3 / 6.8 / 1.9	93.9 / 4.8 / 1.3	< 0.05
Your voice has become hoarse.	82.0 / 13.4 / 4.6	81.0 / 14.5 / 4.5	0.74
You can talk well.	54.4 / 34.6 / 11.1	52.6/36.3/11.0	0.63
Sometimes choking on tea or soup.	86.1 / 13.9	88.6 / 11.4	0.06
You may choke during meals.	75.6 / 18.7 / 5.6	77.4 / 16.8 / 5.8	0.45
You may find it difficult to swallow food.	88.0 / 9.4 / 2.6	86.9 / 10.0 / 3.1	0.66
**Mastication ability (score range: 3 to 9 points)**	4.02 ± 1.07	4.05 ± 1.13	0.53
Compared to six months ago, it is difficult for you to eat tough food.	85.8 / 14.2	85.7 / 14.3	1.00
Mealtimes are now longer than before.	48.9 / 39.5 / 11.5	47.0 / 40.9 / 12.1	0.60
Sometimes you cannot taste it.	90.8 / 7.3 / 1.9	91.0 / 6.9 / 2.1	0.85
**Comprehensive OHI (score range: 15 to 45 points)**	21.70 ± 3.64	21.38 ± 3.68	< 0.05

^a^Variables are expressed as mean ± SD or percentages (%) at each level (two levels of yes/no, three levels of good/normal/poor, or as indicated in the table) separated by “/”

^b^*P*-values were calculated using the t-test and chi-square test for continuous and categorical nominal variables, respectively, as appropriate

^c^The scores of each OHI were calculated by assigning points as follows: 1) 1, 2, and 3 points for ≥3 times/day, twice/day, and ≤ once/day, 2) 1 and 3 points for yes and no, and 3) 1, 2, and 3 points for good, normal, and poor

In women, Table 2 shows that the proportion of obese participants was 16.3%, and BMI of obese group was 27.8 ± 2.8 kg/m^2^ (*p* < 0.001). The mean age was almost the same in the obese and non-obese group, and no difference was found in the number of remaining teeth and proportions of tooth pain, gum bleeding and gum swelling between the two groups. As with the men, the scores on the two OHIs, “oral self-care habits” and “comprehensive OHI” were higher in obese group rather than in non-obese group, and three questions “tooth brushing frequency”, and “use of inter-dental care device” on “oral self-care habits” and “mealtimes are now longer than before” on “mastication ability” were different between obese and non-obese group (at least *p* < 0.05).

**Table 2:** Characteristics of female study participants (*n* = 2552)

Variables^a^	Obese group	Non-obese group	*p* value^b^
	(*n* = 415, 16.3%)	(*n* = 2137, 83.7%)	
Age (years)	56.0 ± 10.2	55.3 ± 10.7	0.26
Body mass index, BMI (kg/m^2^)	27.8 ± 2.8	20.9 ± 2.1	< 0.001
Lifestyle information			
Physical activity (METs‧h/day)	20.6 ± 13.7	20.3 ± 13.1	0.70
Current smoker (%)	6.3	5.5	0.63
Current habitual drinker (%)	35.9	41.6	< 0.05
Snacking, None / Sometimes / Everyday (%)	12.3 / 59.5 / 28.2	15.5 / 47.3 / 37.2	< 0.001
Dietary information			
Total energy intake (kcal/day)	1609 ± 223	1604 ± 309	0.76
Education level, > 12 / 12 / < 12 years (%)	39.8 / 50.1 / 10.1	40.3 / 52.1 / 7.6	0.21
Receiving medications (%)			
Diabetes	9.4	2.0	< 0.001
Dyslipidemia	24.3	14.8	< 0.001
Hypertension	32.5	13.1	< 0.001
Angina myocardial infarction	2.4	1.0	< 0.05
Cerebral stroke	1.7	1.3	0.65
Information on tooth and gum			
Tooth number (n)	25.2 ± 5.1	25.6 ± 4.7	0.10
Tooth/gum condition, No / Sometimes / Yes (%)			
Tooth pain	60.7 / 24.8 / 14.5	62.4 / 23.1 / 14.5	0.73
Gum bleeding	66.3 / 26.0 / 7.7	69.5 / 22.7 / 7.8	0.34
Gum swelling	75.4 / 17.6 / 7.0	73.7 / 19.0 / 7.2	0.76
**Oral health indices (OHIs) and their composed questions**^**c**^			
**Oral self-care habits (score range: 2 to 6 points)**	3.94 ± 1.27	3.51 ± 1.24	< 0.001
Tooth brushing frequency, ≥3 times/day / Twice/day / ≤once/day (%)	32.0 / 53.0 / 14.9	45.6 / 47.0 / 7.4	< 0.001
Use of inter-dental care device	44.3 / 55.7	55.4 / 44.6	< 0.001
**Oral hygiene (score range: 4 to 12 points)**	5.44 ± 1.57	5.47 ± 1.57	0.69
Worried about dry mouth.	83.1 / 16.9	84.6 / 15.4	0.51
Sometimes food may be left in your mouth.	89.6/7.5/2.9	87.9 / 9.0 / 3.1	0.58
A white moss-like substance is coating on your tongue.	81.7 / 14.0 / 4.3	77.7 / 16.8 / 5.4	0.20
Worried about your own bad breath.	44.6 / 36.9 / 18.6	43.0 / 40.9 / 16.1	0.24
**Oral function (score range: 6 to 18 points)**	7.51 ± 1.81	7.41 ± 1.75	0.29
It is getting harder to rinse out your mouth.	93.7 / 5.1 / 1.2	95.0 / 4.0 / 1.0	0.57
Your voice has become hoarse.	85.8 / 10.8 / 3.4	87.5 / 10.1 / 2.5	0.50
You can talk well.	61.7 / 28.4 / 9.9	59.0 / 31.4 / 9.5	0.48
Sometimes choking on tea or soup.	86.3 / 13.7	87.3 / 12.7	0.63
You may choke during meals.	74.0 / 19.8 / 6.3	75.5 / 20.7 / 3.7	0.06
You may find it difficult to swallow food.	86.5 / 9.2 / 4.3	87.1 / 10.1 / 2.8	0.20
**Mastication ability (score range: 3 to 9 points)**	4.02 ± 1.07	3.93 ± 1.06	0.12
Compared to six months ago, it is difficult for you to eat tough food.	88.4 / 11.6	87.5 / 12.5	0.64
Mealtimes are now longer than before.	44.1 / 43.4 / 12.5	51.9 / 38.2 / 9.8	< 0.05
Sometimes you cannot taste it.	91.3 / 7.2 / 1.4	92.1 / 6.0 / 1.9	0.54
**Comprehensive OHI (score range: 15 to 45 points)**	20.90 ± 3.52	20.32. ± 3.50	< 0.01

^a^Variables are expressed as mean ± SD or percentages (%) at each level (two levels of yes/no, three levels of good/normal/poor, or as indicated in the table) separated by “/”

^b^*P*-values were calculated using the t-test and chi-square test for continuous and categorical nominal variables, respectively, as appropriate

^c^The scores of each OHI were calculated by assigning points as follows: 1) 1, 2, and 3 points for ≥3 times/day, twice/day, and ≤ once/day, 2) 1 and 3 points for yes and no, and 3) 1, 2, and 3 points for good, normal, and poor

Table 3 shows that, in men, adjusted ORs (95% CIs and p for trend) were 1.37 (1.11–1.67, *p* < 0.01) for the highest tertile score of “oral self-care habits” and 1.78 (1.42–2.24, *p* < 0.01) for ≤once/day of “tooth brushing frequency”. There was 1.52 (0.81–2.85, *p* < 0.05) for yes of “it is getting harder to rinse out your mouth”, which was one question composed of “oral function”, but no significant risk of obesity was found for “comprehensive OHI”. The significance of Model 1 remained in Model 2, which changed the criteria for the OHI scores.

**Table 3:** Multivariable-adjusted logistic regression analyses of oral health indices (OHIs) for obesity in men

OHIs and their composed questions		ORs^a^	95% CIs	Model 1^b^			Model 2^c^		
				β	SE	*P*_*trend*_	β	SE	*P*_*trend*_
**Oral self-care habits**	Low	1.00	(Reference)						
	Moderate	1.22	(1.00–1.48)	0.10	0.03	< 0.01	0.05	0.02	< 0.01
	High	1.37	(1.11–1.67)						
Tooth brushing frequency	≥3 times/day	1.00	(Reference)						
	Twice/day	1.33	(1.07–1.65)	–	–	< 0.01			
	≤once/day	1.78	(1.42–2.24)						
Use of inter-dental care device	Yes	1.00	(Reference)						
	No	1.09	(0.91–1.29)	–	–	–			
**Oral hygiene**	Low	1.00	(Reference)						
	Moderate	1.03	(0.83–1.28)	0.01	0.03	0.83	0.003	0.02	0.83
	High	1.03	(0.84–1.25)						
Worried about dry mouth.	No	1.00	(Reference)						
	Yes	1.15	(0.93–1.43)	–	–	–			
Sometimes food may be left in your mouth.	No	1.00	(Reference)						
	Sometimes	0.76	(0.57–1.00)	–	–	0.06			
	Yes	0.80	(0.49–1.29)						
A white moss-like substance is coating on your tongue.	No	1.00	(Reference)						
	Sometimes	0.92	(0.73–1.17)	–	–	0.57			
	Yes	0.95	(0.66–1.37)						
Worried about your own bad breath.	No	1.00	(Reference)						
	Sometimes	1.10	(0.91–1.33)	–	–	0.81			
	Yes	0.93	(0.74–1.19)						
**Oral function**	Low	1.00	(Reference)						
	Moderate	0.85	(0.69–1.04)	0.01	0.03	0.75	0.01	0.02	0.75
	High	1.00	(0.83–1.21)						
It is getting harder to rinse out your mouth.	No	1.00	(Reference)						
	Sometimes	1.53	(1.09–2.15)	–	–	< 0.05			
	Yes	1.52	(0.81–2.85)						
Your voice has become hoarse.	No	1.00	(Reference)						
	Sometimes	0.92	(0.73–1.17)	–	–	0.67			
	Yes	1.00	(0.67–1.47)						
You can talk well.	Yes	1.00	(Reference)						
	Sometimes	0.89	(0.75–1.06)	–	–	0.55			
	No	1.00	(0.77–1.31)						
Sometimes choking on tea or soup.	No	1.00	(Reference)						
	Yes	1.28	(1.00–1.62)	–	–	–			
You may choke during meals.	No	1.00	(Reference)						
	Sometimes	1.16	(0.94–1.44)	–	–	0.35			
	Yes	1.03	(0.73–1.47)						
You may find it difficult to swallow food.	No	1.00	(Reference)						
	Sometimes	0.89	(0.68–1.18)	–	–	0.17			
	Yes	0.74	(0.45–1.22)						
**Mastication ability**	Low	1.00	(Reference)						
	Moderate	1.05	(0.87–1.27)	0.06	0.05	0.30	0.03	0.03	0.30
	High	1.12	(0.91–1.37)						
Compared to six months ago, it is difficult for you to eat tough food.	No	1.00	(Reference)						
	Yes	1.07	(0.83–1.37)	–	–	–			
Mealtimes are now longer than before.	No	1.00	(Reference)						
	Sometimes	1.01	(0.85–1.20)	–	–	0.95			
	Yes	1.00	(0.77–1.30)						
Sometimes you cannot taste it.	No	1.00	(Reference)						
	Sometimes	1.03	(0.76–1.41)	–	–	0.87			
	Yes	0.89	(0.50–1.58)						
**Comprehensive OHI**	Low	1.00	(Reference)						
	Moderate	1.07	(0.87–1.33)	0.02	0.02	0.26	0.01	0.01	0.26
	High	1.12	(0.92–1.36)						

^a^Adjusted for age, physical activity, total energy intake, and tooth number as continuous variables, and current smoker, current habitual drinker, snacking, education level, medications [diabetes, dyslipidemia, hypertension, angina myocardial infarction, and cerebral stroke], and tooth/gum conditions [tooth pain, gum bleeding, gum swelling] as categorical variables

^b^The scores on each OHI in Table 1 were divided into the lowest, moderate, and highest tertiles

^c^The scores on each OHI were re-calculated by assigning 1, 3, and 5 points for three levels of variables, and 1 and 5 points for two levels. The values were then divided into the lowest, moderate, and highest tertiles

Table 4 showed that, in women, adjusted ORs were 2.48 (1.80–3.42, *p* < 0.01) for the highest tertile score of “oral self-care habits”, and 3.06 (2.12–4.43, *p* < 0.01) and 1.46 (1.16–1.82) for ≤once/day of “tooth brushing frequency” and no of “use of inter-dental care device”, respectively. There were 1.43 (1.00–2.06, *p* < 0.01) for yes of “mealtimes longer than before”, which was one question composed of “mastication ability”. A significant risk of obesity was found for “comprehensive OHI” (1.74, 1.28–2.36, *p* < 0.01). The significance of Model 1 remained in Model 2, which changed the criteria for the OHI scores.

**Table 4:** Multivariable-adjusted logistic regression analyses of oral health indices (OHIs) for obesity in women

OHIs and their composed questions		ORs^a^	95% CIs	Model 1^b^			Model 2^c^		
				β	SE	*P*_*trend*_	β	SE	*P*_*trend*_
**Oral self-care habits**	Low	1.00	(Reference)						
	Moderate	1.74	(1.28–2.36)	0.26	0.05	< 0.01	0.13	0.02	< 0.01
	High	2.48	(1.80–3.42)						
Tooth brushing frequency	≥3 times/day	1.00	(Reference)						
	Twice/day	1.67	(1.31–2.14)	–	–	< 0.01			
	≤once/day	3.06	(2.12–4.43)						
Use of inter-dental care device	Yes	1.00	(Reference)						
	No	1.46	(1.16–1.82)	–	–	–			
**Oral hygiene**	Low	1.00	(Reference)						
	Moderate	0.95	(0.72–1.27)	−0.05	0.05	0.27	−0.02	0.02	0.27
	High	0.86	(0.66–1.13)						
Worried about dry mouth.	No	1.00	(Reference)						
	Yes	1.05	(0.78–1.42)	–	–	–			
Sometimes food may be left in your mouth.	No	1.00	(Reference)						
	Sometimes	0.75	(0.49–1.14)	–	–	0.26			
	Yes	0.86	(0.44–1.68)						
A white moss-like substance is coating on your tongue.	No	1.00	(Reference)						
	Sometimes	0.79	(0.57–1.08)	–	–	0.13			
	Yes	0.79	(0.46–1.35)						
Worried about your own bad breath.	No	1.00	(Reference)						
	Sometimes	0.93	(0.72–1.20)	–	–	0.59			
	Yes	1.15	(0.83–1.60)						
**Oral function**	Low	1.00	(Reference)						
	Moderate	1.04	(0.79–1.39)	0.06	0.04	0.17	0.03	0.02	0.17
	High	1.20	(0.92–1.55)						
It is getting harder to rinse out your mouth.	No	1.00	(Reference)						
	Sometimes	1.41	(0.84–2.37)	–	–	0.24			
	Yes	1.23	(0.43–3.52)						
Your voice has become hoarse.	No	1.00	(Reference)						
	Sometimes	1.09	(0.76–1.57)	–	–	0.21			
	Yes	1.54	(0.82–2.90)						
You can talk well.	Yes	1.00	(Reference)						
	Sometimes	0.89	(0.69–1.14)	–	–	0.81			
	No	1.06	(0.73–1.55)						
Sometimes choking on tea or soup.	No	1.00	(Reference)						
	Yes	1.13	(0.82–1.57)	–	–	–			
You may choke during meals.	No	1.00	(Reference)						
	Sometimes	0.98	(0.74–1.31)	–	–	0.19			
	Yes	1.68	(1.02–2.76)						
You may find it difficult to swallow food.	No	1.00	(Reference)						
	Sometimes	0.85	(0.57–1.24)	–	–	0.65			
	Yes	1.48	(0.83–2.67)						
**Mastication ability**	Low	1.00	(Reference)						
	Moderate	1.27	(0.99–1.65)	0.12	0.07	0.11	0.06	0.04	0.11
	High	1.23	(0.92–1.65)						
Compared to six months ago, it is difficult for you to eat tough food.	No	1.00	(Reference)						
	Yes	0.87	(0.60–1.26)	–	–	–			
Mealtimes are now longer than before.	No	1.00	(Reference)						
	Sometimes	1.35	(1.07–1.71)	–	–	< 0.01			
	Yes	1.43	(1.00–2.06)						
Sometimes you cannot taste it.	No	1.00	(Reference)						
	Sometimes	1.28	(0.83–1.97)	–	–	0.64			
	Yes	0.83	(0.33–2.08)						
**Comprehensive OHI**	Low	1.00	(Reference)						
	Moderate	1.75	(1.32–2.32)	0.07	0.02	< 0.01	0.03	0.01	< 0.01
	High	1.74	(1.28–2.36)						

^a^Adjusted for age, physical activity, total energy intake, and tooth number as continuous variables, and current smoker, current habitual drinker, snacking, education level, medications [diabetes, dyslipidemia, hypertension, angina myocardial infarction, and cerebral stroke], and tooth/gum conditions [tooth pain, gum bleeding, gum swelling] as categorical variables

^b^The scores on each OHI in Table 2 were divided into the lowest, moderate, and highest tertiles

^c^The scores on each OHI were re-calculated by assigning 1, 3, and 5 points for three levels of variables, and 1 and 5 points for two levels. The values were then divided into the lowest, moderate, and highest tertiles

## Discussion

This study shows that an increased risk of obesity was related to not only oral self-care habits but also oral function and mastication ability as possible, and “comprehensive OHI”, especially in women, among middle-aged and older Japanese men and women. Of the five OHIs and 15 questions on oral health, increased risks were associated with the highest scores for oral self-care habits and poor levels of “toothbrushing frequency” in men and women. In the men, although a positive association was not identified on the “comprehensive OHI”, the increased risks were related to poor levels of the two questions relating to oral function. In women, increased risks were related to the highest scores of “comprehensive OHI” and mastication ability.

Daily oral health care for the health of the whole body is important because poor levels of each of the four OHI (oral self-care habits, oral hygiene, oral function, and mastication ability) have been reported to affect dietary habits and nutritional conditions as well as increase the risk of cardiovascular diseases, diabetes, and cancer [32–34]. In Japan, a national survey of dental diseases in 2016 reported that women were more active in oral hygiene with the use of dental floss and interdental brushes [15]. The number of people who brushed their teeth twice or more every day increased from 16.9% in 1969. Those with toothache and bleeding gums were more likely to be middle-aged, whereas those with chewing difficulties were more likely to be older. Even if those with dry mouth due to diabetes were excluded, dry mouth was found to be more common in middle-aged and older people. For an increased risk of obesity, in the current study, our findings on oral self-care habits in men and women were supported by results from previous studies [14, 35], but we might not have found significant associations with oral hygiene. Surprisingly, a variety of OHIs and a series of oral health questions (e.g., our 15 questions) including “tooth brushing frequency” have not been examined in relation to risks of obesity, diabetes, cardiovascular diseases, and hypertension in many previous studies in Japan and American-European countries.

Unlike men, increased risk of obesity was found corresponding to the highest scores of “comprehensive OHI” and mastication ability, whereas the poor level of the composed question “mealtimes are now longer” for the latter in women. As described above, sex differences have been reported in oral health [36], especially oral hygiene, but not in mastication ability [15]. It was thought that the risk had not been examined for a decreased mastication ability, which was observed with longer meal times. An increased risk of obesity was thought to be associated with a faster meal time rather than slower meal times, considering our previous study on fast eating rate and obesity risk [17]. In other respects, female sex hormones are thought to be related to sex differences because they affect fatty cells to increase body fat, and periodontal tissue and saliva flow to cause tooth decay [37, 38]. Although different oral microbiomes may also be related to the prevalence of periodontal diseases in men and women [39], the details of obesity risk have not been clarified. In the current study, however, no effect of female sex hormones was found on associations with each OHI and the respective question of oral health for the risk, according to the ORs adjusted for menstrual status in women (data not shown). Regardless, in women, the increased risk of obesity was related to the highest score of “comprehensive OHI”, which was systematically composed of the four OHIs including oral self-care habits and mastication ability.

This study had several limitations. First, validity and reproducibility tests of an oral health questionnaire with 15 questions have not been appropriately reported, and a scoring method for the questionnaire has not yet been established to assess oral health. However, in several municipalities, this questionnaire has been used to help plan health projects for residents [29]. We here checked the reproducibility of our findings (i.e., positive or negative β values and their significances) according to the two scoring models used the two ordinal numbers, “1, 2, and 3” and “1, 3, and 5” for each question, respectively. Second, based on the Japanese health insurance system, a questionnaire on oral health was developed for people aged > 75 years in later stages of life. It is important to improve oral health problems in the younger generation because oral health affects diet, nutrition, and even obesity. Third, all data on oral health were self-reported and did not include clinical data such as the depth of periodontal pockets and the presence of dental caries. In the current study, tooth number and tooth/gum condition (i.e., tooth pain, gum bleeding, and gum swelling) were included in the other questions and were used as adjusting factors in the analyses.

Fourth, adjusting for factors may not be appropriate for obesity risk. The risk of obesity has been adjusted for physical activity, but not total energy intake, in many previous studies [30, 40, 41]. In the current study, the two variables were used in the statistical models. Fifth, a cause-result relationship (i.e., “causal reversal”) was not determined in this cross-sectional study using baseline data from a cohort study. We plan to assess the relationship between oral health and obesity risk using data from the same questionnaire administered 5 years after baseline. Sixth, the study participants were not representative of the entire Japanese population. Children and young adults were not included as the target population. However, our study was designed as a population-based study that aimed to assess people with annual health checkups, but not residents, in our study area. Finally, this study did not examine the relationship between dietary intake of foods and nutrients and OHIs on obesity risk, and further research is needed.

## Conclusion

In conclusion, we demonstrated that the increased risk of obesity was related to various OHIs (high scores of "bad" with “good” as reference), that is, “oral self-care habits” for both genders, “oral function” as a possibility for men, “mastication ability” as a possibility for women, and “comprehensive OHI” for women, among middle-aged and older Japanese men and women. Therefore, for the risk of obesity, the current study suggests that it is important to comprehensively assess oral health with reference to WHO’s oral health, but not specifically limited OHIs and their related questions, and that OHIs were shown to be related to poor oral health among middle-aged and older inhabitants. Further studies such as cohort and intervention studies should examine cause-effect relationships between with a various OHIs including “comprehensive OHI” for the risk of obesity.

## Data Availability

The datasets used and/or analysed during the current study are available from the corresponding author on reasonable request.

## Abbreviations

OHI: Oral health index
BMI: Body mass index
OR: Odds ratio
CI: Confidence interval
WHO: World Health Organization
NCD: Non-Communicable Disease
J-MICC Study: Japan Multi-Institutional Collaborative Cohort Study
FFQ: Food Frequency Questionnaire
